# The Resonating Arm Exerciser: design and pilot testing of a mechanically passive rehabilitation device that mimics robotic active assistance

**DOI:** 10.1186/1743-0003-10-39

**Published:** 2013-04-18

**Authors:** Daniel K Zondervan, Lorena Palafox, Jorge Hernandez, David J Reinkensmeyer

**Affiliations:** 1 Department of Mechanical and Aerospace Engineering, University of California at Irvine, Irvine, USA; 2 Instituto Nacional de Neurología y Neurocirugía, Mexico City, Mexico; 3 Department of Anatomy and Neurobiology, Department of Biomedical Engineering, University of California at Irvine, Irvine, USA

**Keywords:** Arm rehabilitation, Stroke, Active assistance, Resonance

## Abstract

**Background:**

Robotic arm therapy devices that incorporate actuated assistance can enhance arm recovery, motivate patients to practice, and allow therapists to deliver semi-autonomous training. However, because such devices are often complex and actively apply forces, they have not achieved widespread use in rehabilitation clinics or at home. This paper describes the design and pilot testing of a simple, mechanically passive device that provides robot-like assistance for active arm training using the principle of mechanical resonance.

**Methods:**

The Resonating Arm Exerciser (RAE) consists of a lever that attaches to the push rim of a wheelchair, a forearm support, and an elastic band that stores energy. Patients push and pull on the lever to roll the wheelchair back and forth by about 20 cm around a neutral position. We performed two separate pilot studies of the device. In the first, we tested whether the predicted resonant properties of RAE amplified a user’s arm mobility by comparing his or her active range of motion (AROM) in the device achieved during a single, sustained push and pull to the AROM achieved during rocking. In a second pilot study designed to test the therapeutic potential of the device, eight participants with chronic stroke (35 ± 24 months since injury) and a mean, stable, initial upper extremity Fugl-Meyer (FM) score of 17 ± 8 / 66 exercised with RAE for eight 45 minute sessions over three weeks. The primary outcome measure was the average AROM measured with a tilt sensor during a one minute test, and the secondary outcome measures were the FM score and the visual analog scale for arm pain.

**Results:**

In the first pilot study, we found people with a severe motor impairment after stroke intuitively found the resonant frequency of the chair, and the mechanical resonance of RAE amplified their arm AROM by a factor of about 2. In the second pilot study, AROM increased by 66% ± 20% (p = 0.003). The mean FM score increase was 8.5 ± 4 pts (p = 0.009). Subjects did not report discomfort or an increase in arm pain with rocking. Improvements were sustained at three months.

**Conclusions:**

These results demonstrate that a simple mechanical device that snaps onto a manual wheelchair can use resonance to assist arm training, and that such training shows potential for safely increasing arm movement ability for people with severe chronic hemiparetic stroke.

## Introduction

The human motor system retains substantial capacity for plasticity following neurological injuries such as stroke and spinal cord injury, and thus intensive rehabilitation exercise can reduce long term motor impairment of both the upper and lower extremities [1-4]. However, rehabilitation exercise delivered one-on-one with a therapist is expensive. There has thus been a rapid surge in the development of robotic and computer-based devices for partially automating intensive rehabilitation exercise [5]. While practice with such devices reduces arm impairment, the devices are still relatively expensive and complex, making them impractical for widespread use. In addition, the viability of using devices that can actively apply large forces to limbs in minimally supervised environments, such as at home, is still unclear.

Developers of rehabilitation technology have previously noted the worldwide need for very simple and effective rehabilitation devices for both assistance and therapy. For example, several organizations have developed low-cost wheelchairs for mobility assistance [6]. Provision of a wheelchair can enhance independence and reduce pressure sore frequency [7]. However, there are relatively few simple and effective technologies to help people with severely weakened arms and hands to engage in therapy for their arms on their own, because most existing equipment requires the ability to grip and/or to move the arms adequately to perform a desired task. This is an important gap to fill because therapeutic arm exercise following neurologic injury can improve arm function and help prevent secondary complications such as contractures [1-4]. If a person regains enough arm movement, then he or she may use the limb more frequently in daily life, further training the limb in a positive cycle, whereas if arm function stays below a threshold, a person may not use the limb, and function may decline [8]. From a pragmatic viewpoint, regaining enough arm strength to push a wheelchair could substantially improve independence.

People with arm weakness can exercise their arms without technology, but if their arms are severely impaired, such exercise is difficult and compliance with autonomous exercise programs is low. Robotic therapy devices have been designed to provide “assistance-as-needed” to arm movement, mimicking the clinical technique of active assisted exercise [9]. Active assistance requires that the patient actively contributes to the movement, a feature of training important for motor learning and plasticity [10]. Active assistance also allows patients with a high level of impairment to participate meaningfully in therapy by limiting frustration, increasing motivation, and promoting self-efficacy. Active assistance may also enhance sensory input that drives motor plasticity [11], and it can demonstrate correct movement patterns that enable better learning [12]. Robots allow a variety of forms of active assistance to be provided for arm training, and, coupled with computer games, can automate training. However, again, robotic therapy devices are typically expensive and complex [5], limiting their widespread use.

The goal of our project was to develop and test in a pilot study a device that could provide active assistance for arm training for people with severe to moderate stroke, but that was also simple and did not rely on powered actuators. We had previously developed an arm therapy device, T-WREX [13], now sold as ARMEO-Spring, which made use of a spring-balanced arm support rather than robotics to assist arm movement. However, while effective in initial studies with people with stroke [14] and multiple sclerosis [15], ARMEO-Spring is still expensive because of the sophisticated counterbalancing and link adjusting system, and because of the use of sensors and a computer for feedback.

The device described here is based on two key concepts. The first concept is to use resonance to assist movement. This concept was inspired in part by a previous study that found substantially improved, long-term recovery of arm movement ability when stroke patients rocked themselves in a rocking chair with their impaired arm, which was placed in an air splint, during subacute rehabilitation [16,17]. Computer algorithms have previously been developed for robotic devices to provide assistance for rhythmic movements [18,19]. However, a passive resonant system accomplishes this goal as well: such a system oscillates with a larger amplitude when it is pushed at its resonant frequency because it stores and releases energy in a manner synergistic to the ongoing movement. A passive resonant system will not move unless pushed, fulfilling the requirement that the exercise be “patient active”. Thus, resonance provides a possible way for weakened patients to amplify their movements, while still maintaining a causal relationship between amount of effort and size of the resulting movement.

The second concept was to integrate the resonant system with an existing, ubiquitous piece of rehabilitation equipment: a manual wheelchair. Many people with arm impairment after stroke or spinal cord injury use wheelchairs, and it is common for people with a neurological injury to spend substantial time in a manual wheelchair during rehabilitation. In addition, as mentioned above, several low-cost wheelchairs have already been developed for use in resource-poor conditions. Our strategy was to reversibly convert a manual wheelchair into a therapeutic technology for the severely weak arm, essentially dual-purposing the chair so that it can be used as an exercise device and then quickly converted back to a mobility aid. This strategy has the advantages of convenience, accessibility, portability, lower net cost and reduced need to transfer the patient to another device. Use of a manual wheelchair also provides a low-friction, high mass base (because of the combined weight of the user and chair), which is ideal for achieving a system with a resonant frequency within a physiologic range.

**Figure 1:**
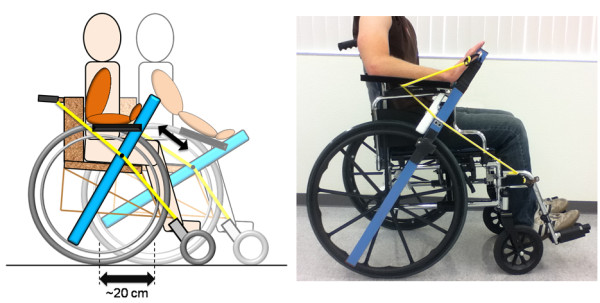
**Left: A schematic drawing of RAE detailing the movement of the various components during operation (the elastic bands that support the arm trough are excluded for clarity, but can be seen in the photo to the right).** The participant uses RAE by pushing rhythmically on the lever in the parasagittal plane, rolling the wheelchair about 20 cm back and forth on the floor at its resonant frequency. The front wheels are rigidly fixed parallel to the rear wheels, so the wheelchair does not rotate in and out of the plane of the figure during operation. Right: A participant’s right arm placed with a “flat-palm” grip in RAE. The elastic bands supporting the arm trough can be seen on both sides of the hand.

We thus created a resonating system by attaching a lever to the wheel of a manual wheelchair and stretching an elastic band from the lever to opposite ends of the wheelchair frame (Figure 1). When the user pushes and pulls on the lever the chair rolls back and forth about 20 cm around a neutral point, storing and releasing energy in the elastic band. We hypothesized that if the user pumped the lever at the resonant frequency of the system then his or her arm’s active range of motion would increase relative to that possible with a single push. Movements with increased range of motion better stretch soft tissue, which may help preserve the suppleness of the soft tissue and reduce spasticity [20], and may also provide somatosensory stimulation that aids use-dependent plasticity [11]. Further, helping people with severe impairment create movements with an increased range of motion may provide a greater sense of self-efficacy, which may be important to motivate exercise by people with a severe motor impairment [21].

This paper describes the design of RAE and experimental results testing the hypothesis that the mechanical resonance of RAE can increase the arm range of motion that the user achieves during exercise. In addition, we performed a small pilot study to test the hypothesis that regular exercise with RAE would lead to improvements in arm mobility for people with a stable baseline of movement ability following severe, chronic stroke.

## Methods

### Device design

RAE incorporates a one meter long, 5 cm × 5 cm square aluminum tube with a notch on the bottom that allows it to pivot on the push rim of a wheelchair and broom handle clamps screwed to the middle of the tube that snap onto the wheelchair push rim to secure the tube rigidly in place. These clamps are placed on both sides of the device, allowing it to be secured to either wheel of the chair for right handed or left handed exercises. An elastic band is placed in tension along the outside of the chair, stretching between a point on the frame near the back and another by the footrest. When RAE is removed from the wheelchair, the elastic band can be tucked inside the arm rest so that it does not interfere with the normal operation of the chair. When RAE is attached, it can clip onto the band at any point allowing the neutral position of the device (and thus the wheelchair) to be easily adjusted. The band then provides a springy resistance when the user moves RAE away from the neutral position and assistance as he or she moves towards it. A padded plastic trough is hinged off of the main shaft to support a patient’s forearm during therapy. Adjustable elastic bands connected between the main shaft and the forearm trough allow for the level of weight support of the forearm trough to be adapted to each patient. Velcro straps are used to secure the user’s arm in the trough and his hand to the main shaft during exercise. The user can use either a standard grip, in which he or she grips the shaft like a glass of water, or a “flat palm” grip, in which his or her hand is strapped to the shaft with the fingers extended (see Figure 1, right). Movement of RAE requires shoulder flexion/extension, elbow flexion/extension, and wrist flexion/extension; in achieving these movements the arm moves in the parasagittal plane along with the forearm support and lever.

When a user pushes forward on RAE, the wheelchair wheel that RAE is rigidly attached to rolls forward as well about 10 cm, and the rear elastic band is stretched in tension, storing energy (see Figure 1, left). In order to make the entire wheelchair roll forward in a straight line even though it is only being driven by one wheel, the front caster wheels of the wheelchair are clamped so that they are fixed parallel to the rear wheels. Fixing the caster wheels in this manner also reduces the damping ratio of the system, since no energy is lost to the rotation of the front wheels out of the sagittal plane. Keeping the damping ratio low is important to ensure the system is sufficiently underdamped to be resonant. When a user stops pushing forward on RAE, and/or begins to pull backward on RAE, the tension in the rear elastic band assists in returning RAE, and thus their arm, toward the neutral position and then beyond (i.e. more into elbow flexion). This action causes the attached wheel, and thus the whole wheelchair, to roll backward about 20 cm, and it causes the front elastic band to become stretched in tension, once more storing energy in the system. Again, because the front caster wheels are fixed, the wheelchair rolls backward in a straight line through its original position. If the user stops pushing or pulling on RAE, the wheelchair stops rolling and settles around the neutral position. Please note that although the wheelchair is technically rolling forward and backward in a straight line, due to the conceptual background of the device and the similarities of its operation to a rocking chair, we refer to this motion as “rocking” for simplicity throughout the rest of this paper, which is also consistent with a standard dictionary definition of rocking as “gently moving to and fro”. A video of a volunteer using the device can be found at http://youtu.be/jn8ojnfYWQ8.

Because RAE is designed to be resonant, it assists a patient in obtaining a larger range of motion (moving further away from the neutral position) if he or she rocks back and forth at the resonant frequency of the system. To see the theoretical basis of the design, approximate the distributed system of mass, damping, and stiffness as a lumped-parameter, mass-spring-damper system, and assume a person can generate a maximum pushing force on the lever equal to F_max_. Assume the total stiffness of the elastic cords and the user’s arm, acting in the direction of rocking motion of the lever, is K. Then the maximum distance the hand moves when the person pushes with maximum force is:

(1) $$x_{\max}=\frac{F_{\max}}{K}$$

Now, if the system is resonant (i.e. the damping ratio ζ < 0.707), and the person pushes with a force F = F_max_sin(ωt), where ω is the resonant frequency of the system, then the distance the hand moves will be [22]:

(2) $$x_{\max}=\frac{F_{\max}}{K}A,$$

where the “movement amplification gain” A is given by:

(3) $$A=\frac{1}{2\zeta \sqrt{{1-\zeta }^{2}}}$$

This means that if the person still pushes with strength F_max_, but at just the right time, periodically, then the amplitude of the hand movement will grow to be A times larger than is possible with just a single maximum push. Note that A depends on the damping ratio ζ, which is given by the stiffness K (set by the elastic band and biomechanical stiffness of the arm), damping C (set by the friction in the system and the biomechanical damping of the arm), and mass M (i.e. total inertia of the chair and lever, and the person including their body mass and the inertia of their arm) of the system according to:

(4) $$\zeta =\frac{C}{2\sqrt{\mathrm{KM}}}$$

Note that the average amplitude of rocking is proportional to the average force applied to the lever. If the user stops pushing, the device stops rocking; thus the device requires active effort by the user, and the user is rewarded with a larger range of motion if he or she tries harder and maintains the correct movement timing. Note also that it is important for the resonant frequency of the system to be within physiologic range for human movement (~1 Hz) while still providing appropriate range of motion of the arm. The resonant frequency is given by:

(5) $$f=\frac{1}{2\pi }\sqrt{\frac{K}{M}}$$

Importantly, the resonant frequency of RAE is in physiologic range because the mass to be moved is large, as it includes the user’s own mass combined with the mass of the chair as the chair rolls.

### Experimental protocol

We performed two pilot experiments with RAE. The first was designed to test the hypothesis that RAE’s resonant property would amplify the active range of motion (AROM) of a user’s arm. In this experiment, we first measured the step response of RAE with six volunteers with a chronic, severe stroke. To do this, the volunteers were asked to hold RAE but to relax the arm, and the experimenter pulled RAE forward approximately 40 degrees, extending the arm, and then released RAE two times. A tilt sensor (Nintendo®’s Wii Remote) attached to RAE measured the angle change of the device at 20 Hz, and we measured the damping ratio of RAE using the logarithmic decrement method [22]. The sensor was placed 10 cm from the end of the shaft on the bottom side. We predicted the resonant frequency of rocking, ω_res_, for each volunteer from the damped natural frequency of the step response, ω_d_, using the equation [22]:

(6) $$\omega_{\mathrm{res}}{=\omega }_{d}\frac{\sqrt{{1-2\zeta }^{2}}}{\sqrt{1-}\zeta^{2}}$$

We also compared the predicted step response of RAE (i.e. based on second-order, linear, mass-spring-damper model described in the previous section, using the measured damping ratio and the measured damped natural frequency for each participant) to the actual step responses we measured.

To measure the unamplified range of motion, we then asked the six volunteers with chronic stroke to push and hold RAE as far forward as possible with their impaired arm three times, and then to pull and hold RAE as far backward as possible three times. The volunteers were monitored to ensure that they did not lean with their trunk to extend their AROM in the forward direction. To measure the effect of the mechanical resonance, we asked the volunteers to rock RAE at whatever frequency felt natural, again monitoring them to prevent leaning. Our goal was to determine if the volunteers would naturally rock at the resonant frequency, and if the AROM achieved during rocking was greater than that achieved during the isolated, maximum effort push and pull, as predicted by the theory outlined above. Subjects performed informed consent according to the approved procedures of the U.C. Irvine Institutional Review Board.

We also conducted a separate pilot study of RAE with different subjects to provide an initial assessment of its value as a rehabilitation device. The question we were interested in was, “If individuals with a severe chronic stroke, who have finished formal rehabilitation and have reached a plateau of arm ability, exercise with RAE, will they improve their arm movement ability without experiencing an increase in arm pain?” Thus, for this study, we recruited eight volunteers with a stroke from the outpatient population of the Instituto Nacional de Neurología y Neurocirugía in Mexico City, and the volunteers provided informed consent according to the procedures approved by the INNN Institutional Review Board. Inclusion criteria were > 6 months post injury, moderate to severe arm movement impairment defined as an upper extremity Fugl-Meyer (FM) score < 35 out of 66 [23], and willingness to refrain from additional rehabilitation for the upper extremities during the 6 week duration of the study. The average age of the participants was 52 ± 15.

We assigned participants to two groups based on their availability. Participants in the exercise-rest group (n = 3) exercised with the device for 3 consecutive weeks, and then rested for 3 consecutive weeks; participants in the rest-exercise group (n = 5) reversed the order of exercise and rest. Arm mobility typically reaches a plateau in chronic stroke by many measures, provided individuals maintain a relatively steady level of activity [24]. Nevertheless, we used the data from the rest-exercise group to confirm the well-known plateau for this study. The existence of the plateau allowed us to then use the participant’s baseline assessments as the control.

During the exercise period, the participants rocked RAE for a total of six hours in eight forty-five minute sessions spread over the 3 weeks. They were continuously monitored by an investigator to ensure that they did not perform compensatory trunk movements or experience discomfort. We increased the stiffness of the elastic band after 4 sessions for every participant by stretching the band to a more extended operating point; since the band stiffness increased with length, this increased the stiffness of the band. This was done to compensate for the fact that the band we used in this study tended to wear out mechanically at the connection points to the chair.

The primary outcome measure was an automated measure of active range of motion (AROM) of the arm obtained using RAE. We quantified AROM of the arm using an improved tilt sensor (ADXL 213) attached to RAE in the same manner described above. We asked the participants to rock 50 times, and recorded the angle of RAE relative to the initial position at 50 Hz using a microcontroller (PIC 18 F2455). We defined the AROM as the average amplitude of the angle change during rocking. The participants repeated this test three times per session to establish an average for that day. We obtained a baseline AROM measurement for each participant on a separate day before the participants began the exercise period. Then we performed the AROM measurement immediately before each of the eight exercise sessions. This gave us a baseline measurement of AROM for each participant before they began therapy and 8 measurements after therapy began. Secondary measures were the upper extremity Fugl-Meyer score and subjective report of arm pain. The same non-blinded therapist evaluated FM score at the start and end of both the 3 week rest period and the exercise period, and at a 3 month follow-up evaluation. Each participant indicated their arm pain level before and after each session on a visual analog pain scale from 0 to 10, with 0 being no pain, and 10 being the greatest pain possible.

When normality was confirmed, we analyzed changes in the outcome measures using parametric statistics including the t-test. If normality was violated, we used non-parametric statistics.

## Results

### Amplification of Arm movement with resonance

**Figure 2:**
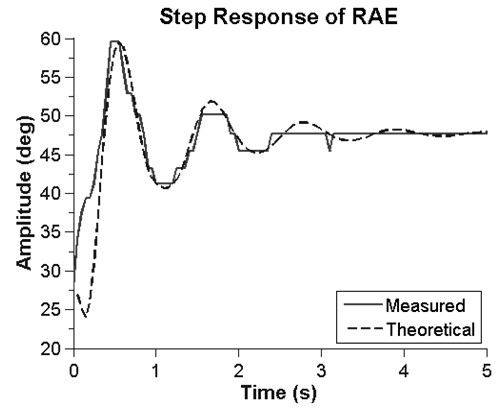
**The step response of a subject in RAE.** The superimposed dotted line shows the theoretical angle an accelerometer would measure during the step response of a second order system parameterized with the experimentally identified damping ratio and natural frequency.

**Figure 3:**
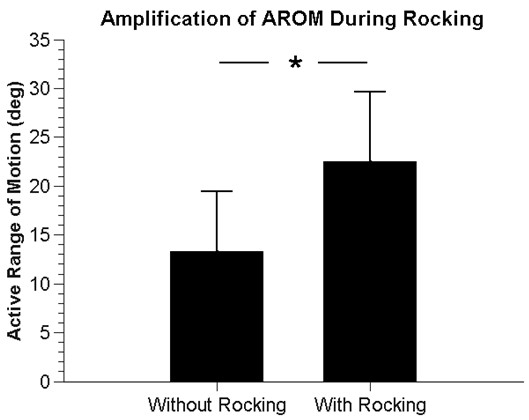
**The arm AROM of individuals with chronic stroke in RAE.** The “without rocking” bar shows the AROM achieved with RAE with a single effort, defined as the difference between a single, sustained maximum push, and a single, sustained maximum pull. The “with rocking” bar shows AROM when participants were asked to rock at whatever frequency felt natural. The amplitude of movement was 1.7 times larger when participants were rocking, a significant difference (p = 0.041).

In a first experiment we tested whether the mechanical resonance property of RAE would amplify arm AROM of participants with stroke (n = 6), as predicted by the theory. First, to verify that RAE acts like an underdamped, linear second order system, we measured the step response of RAE. The step response was well approximated by a second order linear model with a mean RMSE over 12 trials of 1.9 ± 0.6 degrees (Figure 2). The mean damping ratio was determined from the logarithmic decrement method [22] to be 0.2 ± 0.04, which yielded a predicted movement amplification gain of 2.6 if the participants chose to rock at the resonant frequency, according to Equation 3. Indeed, the subjects intuitively rocked at the resonant frequency when we asked them to rock RAE: the resonant frequency of RAE predicted using Equation 6 and data from the step response was 0.88 ± 0.15 Hz, and the actual frequency the patients chose to rock at was 0.84 ± 0.16 Hz, a non-significant difference (t-test, p = 0.5). AROM, defined as the maximum angle change of the device from flexion to extension, increased significantly when the subjects rocked RAE compared to a single push and pull (Wilcoxon test, p = 0.041), and the resulting amplification of the participants’ range of motion was 1.7 (Figure 3).

### Pilot testing as an Arm exercise device after chronic stroke

**Figure 4:**
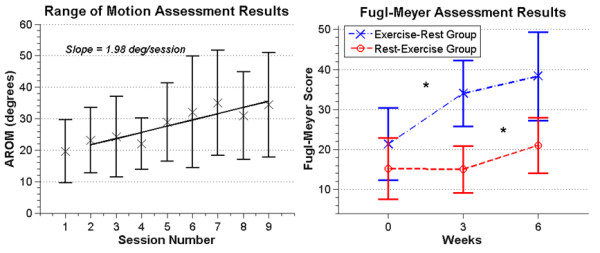
**Left: A plot of the mean AROM for 6 participants (the remaining two participants had full range of motion along the device at study start).** Error bars show +/− 1 SD. Each participant had one baseline measurement and 8 measurements during the exercise period. The solid line is the linear regression showing a positive slope of 2.0 degrees per session (R^2 = 0.80, p = .003). Right: The mean FM scores for the Exercise-Rest (n = 3) and Rest-Exercise (n = 5) groups. Error bars show +/−1 SD. Significant changes are marked with a ‘*’.

In a second pilot study with a different set of eight volunteers with a chronic stroke, we measured the effect of repeated use of RAE on arm movement ability and arm pain for individuals who had ceased formal rehabilitation. The mean initial FM score for the eight participants in the pilot study was 17 ± 8 out of 66 points; i.e. the participants had substantial arm impairment. There was not a significant difference between the initial FM scores for each group (Wilcoxon rank sum test, p = 0.29). The FM score of the Rest-Exercise group did not increase during the rest period (Figure 4), indicating a stable baseline. This was expected for individuals who were on average 3 ± 2 years post-stroke and had severe arm impairment [24].

Average AROM of the arm measured with RAE improved steadily across the three weeks of exercise (Figure 4), with the average data being well fit by a line with a slope of about 2 degrees per session (R^2 = 0.80, p = .003). Note that we excluded two participants from this analysis who had full AROM along RAE at study start. The overall average increase in AROM for the remaining 6 subjects was 14 ± 9.8 degrees, or 66% ± 20%, after three weeks of RAE exercise.

**Figure 5:**
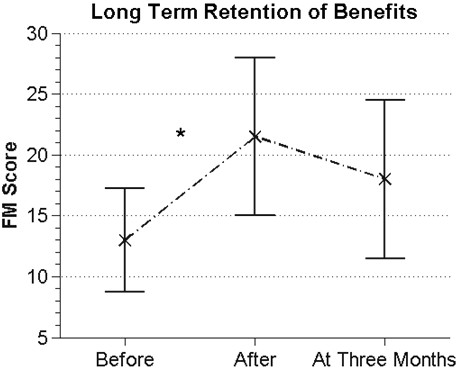
**The median FM scores (n = 6) from before therapy, immediately after therapy, and at a three month follow up assessment.** A significant change in FM score was detected before and after therapy (p = 0.042), but no significant change was detected three months later (p = 0.80), although there was a slight downward trend. Error bars show the interquartile range.

The mean change in FM score after three weeks of exercise with RAE, averaged across all participants (n = 8), was 8.5 ± 4.1 points, while the mean change after the three week rest period for all participants was 1.5 ± 4. This difference was significant (t-test, p = 0.009), with the assumption of normality confirmed for both change distributions (Lilliefors test, p = 0.67 and 0.89, respectively). We hypothesized that the small average improvement in FM score across all subjects during the rest period arose because the group that exercised with RAE first continued to improve during the subsequent rest period. Indeed, the FM score of the Exercise-Rest group (n = 3) increased by 4.3 ± 4.1 points during the rest period (Figure 4) compared to a change in the Rest-Exercise group of −0.2 ± 3.2 points during the rest period (i.e. a stable baseline, n = 5), but this difference was not significant (t-test, p = 0.13). Figure 5 shows improvements in FM score were sustained at the 3 month follow-up for 6 participants. We were unable to obtain follow-up measurements for the other two participants due to loss of contact. Because the sample size was small, we performed a non-parametric test (Friedman test) on the before, after, and three-month FM scores and again found a significant change in median score (p = 0.042); the follow-up multiple comparison test showed no significant difference between the after and three-month scores (p = 0.80).

**Figure 6:**
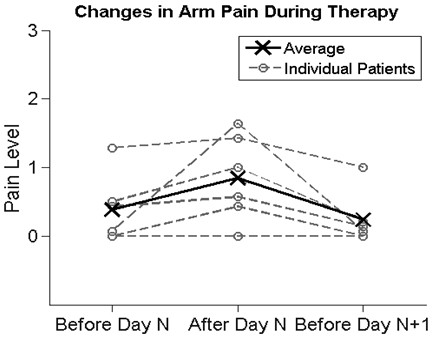
**The results of the pain measurements, showing the average perceived levels of pain before a session, after that session, and before the following session.** The dashed lines represent each individual participant and the solid line represents the mean values for all 8 participants.

Participant rating of arm pain increased slightly by a non-significant amount (p = 0.11) at the end of each exercise session relative to the beginning, but returned to approximately its starting value by the next session (Figure 6).

**Figure 7:**
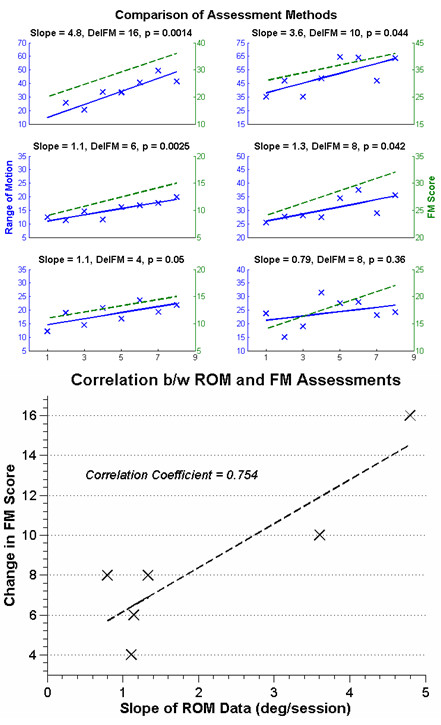
**Above: Comparison of the FM and AROM assessments for 6 participants (the remaining two participants had full AROM along the device at study start).** The solid line represents the regression line for the AROM data, while the dashed line shows the change in FM score before and after training. Below: Comparison of the slope of the AROM data vs. the change in FM score for 6 participants (the remaining two participants had full range of motion along RAE at study start). The dashed line is an estimate of a linear relationship between the two measurements (R = 0.75, p = 0.09).

We analyzed whether the changes in AROM correlated with the changes in FM score. This analysis was done for the same six participants included in the AROM analysis above (i.e. those 6 who could not push RAE to its full range of motion). One of the data sets did not show significant change in AROM, but it was still included for completeness (Figure 7). The slopes of the lines fit to the increases in AROM for each subject moderately correlated with their FM score changes (Spearman correlation, R = 0.75, p = 0.09).

Using the mean frequency from the AROM data of 0.87 Hz and an exercise period of about 40 minutes, we estimated that the participants performed about 4000 movements per session (2000 flexions and 2000 extensions). This assumes that the participants rocked continuously in each session, with no breaks for the entire 45 minute session, which was verified by the investigator who continuously monitored each session. This adds up to roughly 32,000 practice movements with a specific, intentional timing performed by each participant over the eight exercise sessions.

## Discussion

We developed a simple device called RAE that allows people with substantial arm weakness to practice arm movement while receiving mechanical assistance for that movement. RAE snaps onto a manual wheelchair push rim, turning the wheelchair into a resonant system that can be gently moved to and fro by pushing on RAE. The resonance of the system amplifies arm AROM, as we verified experimentally here. We also found that people with a chronic stroke who trained with RAE for three weeks significantly improved their arm movement ability over their stable baseline, as measured by both an objective assessment of AROM and the Fugl-Meyer score. Additionally, a moderate correlation was found between these two measurements, suggesting that the device itself may provide a simple means of measuring motor function that is comparable to an established clinical measurement, although this would need to be verified in a larger study since the observed correlation was dependent on two subjects who recovered relatively well. Importantly, subjects did not report discomfort during rocking or an increase in arm pain with repeated use of RAE. We first discuss the mathematical operating principle of RAE and then the results of the pilot testing of arm training with RAE.

### Mathematical operating principle of RAE

**Figure 8:**
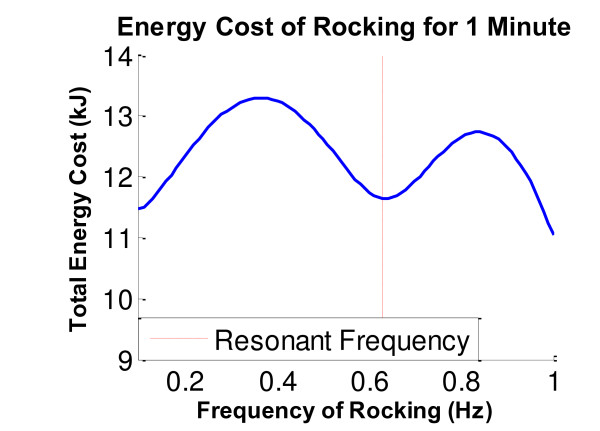
**Theoretical energy cost of rocking in RAE.** The energy cost for each rocking frequency was computed using a mathematical model of human energy expenditure [25] for a human driving a second-order underdamped system with the same damping ratio and natural frequency identified in Figure 2 above. Using this model, we calculated the instantaneous rate of energy expenditure as the sum of the velocity and force dependent heat losses in the muscle and the rate of mechanical work being done, then integrated this rate over a one minute time span to calculate total energy expenditure.

To model RAE and analyze how its resonance properties amplified the subject’s movement attempts, we made several assumptions. First, we assumed that the distributed system of mass and stiffness created by the user and RAE could be modeled using lumped elements operating on the lever. Second, it is well known that muscle damping changes with activation (i.e. Hill’s relationship), but we assumed that these changes were relatively small, and that the system behaved like a second order, linear, under-damped system. Third, we assumed that the individuals we tested rocked the system with a sinusoidal torque near the resonant frequency. The experimental data we collected verified that RAE indeed closely approximated the response of a second order linear system driven with a sinusoidal force, and that the individuals we tested rocked at a frequency very near the predicted resonant frequency. It is likely that the subjects rocked so close to the predicted resonant frequency due to the reduced effort required to rock at a given amplitude near the resonant frequency. Figure 8 shows a theoretical plot of the energy required to rock for one minute at various frequencies; a local minimum is clearly present at the resonant frequency due to the amplification of the user’s input during resonance. The measured value of the amplification of the individuals’ AROM was 1.7, less than the predicted value of 2.6. This was likely due to increases in muscle- and/or reflex- related damping of the arm when the muscles were activated during rocking. Thus, the simple second order linear model provides an adequate conceptual basis to understand the operation of the system. Essentially, RAE amplifies movement because it is like a mass-spring-damper system being forced at its resonant frequency by the user’s efforts. User’s find the resonant frequency easily likely because it takes less effort to rock at that frequency, for a given amplitude of arm movement.

### Interpretation of pilot testing of Arm training with RAE

The pilot therapeutic study showed that chronic stroke subjects with a stable baseline who rocked RAE improved their AROM, reduced their arm impairment, and did not experience an increase in arm pain. It is highly unlikely that these results are the result of a “placebo” effect as the improvements in AROM accrued gradually with training. It is also highly unlikely that these improvements arose from increased use of the arm outside of RAE, as the subjects who participated had low FM scores, which has been shown to correlate strongly with low participation in activities of daily living [26].

A limitation of this pilot therapeutic study was however the small population size (i.e. this was a pilot study only). Thus, while the training effects were highly significant here, their magnitudes should be estimated with higher power using larger studies. In addition, we used a non-blinded evaluator for the Fugl-Meyer assessments. This limitation is somewhat offset by the moderate correlation between the FM scores and the objective AROM data. Another issue of concern is whether exercising with the device would increase spasticity or cause spastic movements. Although we did not measure spasticity, we did not observe any spastic movements. Other researchers have found that high resistance strength training actually commonly reduces rather than increases spasticity after stroke [3]. The arm exercise done with RAE can be viewed as a form of low-to-moderate intensity resistance exercise, and thus would also not be expected to increase spasticity based on this previous research.

The question we designed the pilot testing of arm training to answer was, “If individuals with a severe chronic stroke, who have finished formal rehabilitation and have reached a plateau of arm ability, exercise with RAE, will they improve their arm movement ability without experiencing an increase in arm pain?” We felt this was the most important question to answer at this stage of device development, because it provides the needed basis for future studies comparing the efficacy of RAE with other therapeutic techniques. That is, given that subjects did not exhibit an increase in arm pain and did indeed improve their AROM, future studies with a larger population size should now be conducted to compare the effect size of RAE-based therapy to other standard therapies. In addition, this study provides preliminary insight into the pragmatic question of whether adding a program of rocking on top of their existing activities is useful for individuals with chronic stroke.

The improvements in AROM and FM score that were observed after using RAE were relatively large compared to many previous robotic therapy studies (e.g. compare ~3 point gains for the recent multi-site study of MIT-MANUS [27] and T-WREX [14]). As mentioned above, this was a pilot study only, and the effect size may decrease with larger numbers of subjects. Nonetheless, we believe the principle of using mechanical resonance to mimic robotic assistance of the weakened arm is a promising approach for several reasons. As explained above, resonance requires that the patient be active to keep the system moving, and requires “goal directed” movement, as it penalizes movements that are not precisely timed by resisting them. Resonance also provides assistance comparable to that provided by “assist-as-needed” robotic therapy devices: external forces are applied to the arm, and the amplitude of the resulting movement is proportional to the force applied but also to the effort of the patient. In addition, for both RAE and assist-as-needed robotic therapy devices, the amount of assistance is tunable by altering system parameters (stiffness and damping and therefore the movement amplification gain). Programmable resonance has been proposed previously as a method to hide the inertia of a gait robot [28], and a robotic-assistance algorithm that assists rhythmic movement by adapting to the frequency of a user’s movements has been proposed as a method of assisted rehabilitation [18].

Further, if one hypothesized that performing the most number of active repetitions possible in a given time is best for promoting recovery, then working with a resonating system is a good way to achieve many repetitions. As stated above, each participant performed roughly 32,000 movements during the study. These movements require timing and effort, and they are “goal-oriented”, in the sense that they are chosen to sustain rocking. They can also be viewed as emulating the motions needed to perform a reach-to-grasp movement (shoulder flexion and elbow extension), and are thus “functional” or at least reasonable precursors to a key function in some sense. We speculate that the sheer number of these timed, effortful, goal-directed movements that were practiced may have contributed to the observed, robust recovery the study participants had with RAE.

Also, as mentioned in the introduction, a previous study used a rocking chair as an arm therapy tool in a large number of stroke patients with similarly strong results, although that study was done soon after stroke [16,17]. The participants rocked themselves with their impaired arm, which was braced in an air splint. A control group was rocked by a caregiver, but did not actively use the arm. The rocking amount was matched between the groups. The subjects who actively rocked themselves improved their Upper Extremity FM score by 17 points more than the control group at the 5 year follow-up (n = 62). This is a substantial, clinically significant difference, exceeding improvements found with much more complex technology, and is consistent with the effects we observed with RAE.

Although RAE is a resonant system like the rocking chair, and thus may have similar therapeutic benefits, it has several differences that may be useful. RAE uses a lever to increase the active range of motion of shoulder flexion/extension, elbow flexion/extension and wrist flexion/extension used for training, while rocking a rocking chair in an air splint requires smaller joint movements. In addition, RAE is easily adjustable; i.e. the resonant frequency and amount of amplification of arm movement of RAE can be adjusted by changing the elastic cord stiffness and damping, which is not possible with a rocking chair.

Other simple devices have been developed that require rhythmic motion of the hand along a ramp or slide, in a motion somewhat similar to that used with RAE. For example, the BATRAC provides auditory cueing of rhythmic arm movements on a track using a metronome [29-31]. RAE is different in that it provides mechanical assistance using resonance, but similar in that both devices require the users to try to time their movements. BATRAC does this explicitly by providing an auditory cue. RAE does this implicitly because pumping RAE at a frequency other than the resonant frequency requires greater effort. We plan to explore the differences between explicit and implicit feedback for motor recovery in future studies.

Another useful feature of RAE is that it can be attached to a wheelchair the patient is already using, rather than necessarily requiring a transfer. The design of RAE could also be extended to include visual or audio feedback as a motivational tool for patients and as a real-time assessment tool for therapists.

## Competing interests

Daniel Zondervan and David Reinkensmeyer have a financial interest in Flint Rehabilitation Devices, LLC, a company that develops rehabilitation devices. The terms of this arrangement have been reviewed and approved by the University of California, Irvine, in accordance with its conflict of interest policies. The remaining authors declare that they have no competing interest.

## Authors’ contributions

DZ performed the design and construction of the RAE device, conducted both pilot experiments and drafted the manuscript. LP performed the Fugl-Meyer assessments and monitored the participants in the therapeutic pilot study for adverse events. JH provided collaboration to make the therapeutic pilot study in Mexico possible and consulted on the study design. DR inspired and collaborated on the design of the RAE device, participated in the design and coordination of both studies, and assisted in drafting the manuscript. All authors read and approved the final manuscript.
